# Dysfunctional Cellular Energy Metabolism is a Premorbid Characteristic of Late‐Onset Alzheimer's Disease

**DOI:** 10.1002/alz70855_098691

**Published:** 2025-12-23

**Authors:** Kai Christian Sonntag, Bruce M Cohen

**Affiliations:** ^1^ McLean Hospital, Harvard Medical School, Belmont, MA, USA

## Abstract

**Background:**

Age is the main risk factor for late‐onset Alzheimer's disease (LOAD) and all processes in normal aging also occur in LOAD pathology. While some factors appear to be more specific to LOAD and may be genetically determined, others are acquired through the aging process. Because bioenergetic metabolism is among the most fundamental features of cell functions, changes thereof have profound effects on every aspect of aging and could be key determinants in the pathology of LOAD.

**Method:**

We developed a cellular platform for LOAD collecting skin fibroblasts or blood cells from LOAD patients and healthy control individuals that are used in the induced pluripotent stem cell (iPSC) paradigm to produce brain cells for determining LOAD pathogenic processes in context of age, disease, genetic background, cell development, and cell type. Changes in bioenergetic and metabolic cell functions are evaluated using a battery of molecular and biochemical assays.

**Result:**

In both fibroblasts and iPSC‐derived immature and mature brain cells of subjects with LOAD, we observed bioenergetic substrate deficiencies, including reductions in the redox agent nicotinamide adenine dinucleotide (NAD) or glucose uptake and metabolism, and alterations of associated bioenergetic‐dependent cell functions.

**Conclusion:**

Overall, our data suggest that dysfunctional bioenergetics is an inherent cell‐specific and cell‐autonomous risk factor in LOAD. Together with findings in the brains of normally aging individuals or in patients with LOAD, the data support the view that in LOAD, inherent cell‐metabolic dysfunctions may occur in development and early life altering the trajectory of the normal aging process already in youth and middle‐age and predispose neuropathological events that lead to symptomatic features of LOAD in late life. Our studies seek to further identify and characterize the cellular mechanisms underlying LOAD‐associated bioenergetic abnormalities. Results of these studies may reveal targets and methods for treating individuals at risk for LOAD before illness develops or delaying disease onset and progression later in life.

